# A Fresh Evidence of Income Inequality and Health Outcomes Asymmetric Linkages in Emerging Asian Economies

**DOI:** 10.3389/fpubh.2021.791960

**Published:** 2021-12-23

**Authors:** Shuangshuang Chang, Bin Gao

**Affiliations:** ^1^School of Business, Wuchang University of Technology, Wuhan, China; ^2^School of Economics, Guangxi University for Nationalities, Nanning, China

**Keywords:** income, inequality, health, Asian, economies, ARDL, PMG

## Abstract

During the last few decades, income inequality in emerging Asian economies has been increased dramatically. It is widely recognized that income inequality has severely impacted population health. This study attempts to estimate the impact of income inequality on health outcomes in emerging Asian economies for a time horizon ranging from 1991 to 2019. Our empirical analysis shows that income inequality has a negative effect on life expectancy in the long run. We also find that positive changes in income inequality decrease life expectancy, but a negative change in income inequality increases life expectancy in the long run in emerging Asian economies. The symmetric and asymmetric results are robust to different measures of econometric methods. Thus, governments should pay more attention to the consequences of their economic policies on income inequality to improve health outcomes.

## Introduction

To be healthy is the primary concern of any individual because, without health, a person cannot play an active role in any walk of life. If a person remains unhealthy for a longer time, he cannot take care of himself, his family and become a liability for society. On the other side, a healthy mind and body can contribute to the socioeconomic development of society in many ways; thus, it becomes an asset for society. However, deciding whether a person is healthy or unhealthy is difficult because health is not just the absence of illness but much more. Wolinsky and Arnold (1) recommended that health should be regarded as a complex and multidimensional framework. It is the absence of physical diseases and is a sign of active participation in society and an indicator of psychological health status. He then classified each person based on each indicator, either “well” or “ill.” Barr (2) extended the work of Ware and Sherbourne (3) by postulating that corporal health should be categorized not just as the non-existence of signs, but by the degree of disease or discomfort, and mental health by the fluctuating grades of psychological health situations like depression and Alzheimer's disorder. Likewise, he proposed that behavioral well-being should be categorized from the point of view of an individual's healthy (regular exercise) or non-healthy (smoking) behaviors and the individual's capability to work in society and accomplish day-to-day tasks. Such a complex model could prove as a foundation for examining the relationship between health and income inequality.

Literature on health and social science is filled with the growing number of studies with regard to the effects of income, poverty, and social policies on a person's physical and mental well-being (4, 5). Several studies are available in the literature which supports the notion that the socioeconomic well-being of the individuals Wanberge et al. (6) and Soldevila-Domenech et al. (7) or its related factors such as income Niessen et al. (8), education Montez et al. (9), and occupation Álvarez-Fernández (10) significantly affect the person's health condition. These studies are noted that social as well-economic factors are more important for health. In the middle of the 1970s, researchers started to raise questions on the effects of the national income on people's health within advanced economies (11). It was observed that when a county achieved a particular level of economic development, from that point onwards, any additional rise in income did not contribute much to increasing the life expectancy at the national level (11). The epidemiological evolution theory describes two different types of outcomes. First and foremost, a shift will occur in epidemiology, i.e., more deaths will be caused by chronic diseases instead of infectious diseases. Secondly, the life expectancy will increase at a national level because more people will die at old age, and the mortality rate of young people will decline (12).

Though several studies in the past have focused on the health status of the people within different countries Mahasneh et al. (13), however, in the year 1980, when Britain's Black Report came to fore which published material related to health inequalities, the researchers started to focus more on the relationship between health and income inequalities across various social groups and less on the effects of aggregate income on the overall health status of the people (14). Accordingly, studies on differences in health conditions amongst persons with diverse socioeconomic positions have exaggerated over the last few decades. Tibber et al. (15) noted that income inequality is negatively associated with mental health and infers that income inequality contributes significantly to mental health problems. While Qasim et al. (16) depicted that income inequality is adversely associated with human development. The impact of income inequality on health is also significant, and poverty is also considered the main contributor to health problems (17).

After the economic crisis of the year 2008, the awareness related to the consequences of income inequality increased in the USA and all across the globe. These concerns were not baseless; instead, based on the fact that during the period 2009–2012, there was a 31.4% rise in income of the richest 1% of people against 0.4% of the bottom 99% people (18). The consequences of such a great economic disproportion cause anxiety for any country's confidence and sense of justice and have serious repercussions for the nation's health. Therefore, academics, policymakers, and health economists focused on the nexus between income inequality and health status. There is consensus among the researchers that the low income or socioeconomic status has a negative impact on the health conditions of the people. Consequently, income inequality has become an important determinant of the health status of the people. Economic disparity has been revealed to exert extra stress and abridged social capital facilities for the people who belong to deprived or low-income classes.

Besides, these people are also not highly educated, which repercussions an individual's health (19). As the gap between rich and poor widens, the gap between their socioeconomic status and related indicators such as education, health, the living standard also widens and the magnitude of the problem is more pronounced in developing and emerging economies (20). Therefore, income inequality is a more serious concern for developing and emerging economies than developed economies; thus, the negative effects of income inequality on health outcomes could be more severe for developing and emerging economies. Moreover, the health structure in such economies is not as advanced as in the developed economies; hence, income disparities in such economies could seriously disrupt the health status of the people. Therefore, answering how income inequality affects health outcomes in emerging Asian economies has become a pertinent question. The emerging Asian economies also have an issue of income unequal. Baek and Shi (21) reported that income inequality has significantly increased with the level of globalization in emerging economies. This study motivates us to assess the impact of income inequality on health in emerging Asian economies and the transmission channels in this process. This study differs from the earlier studies in one important way. This study explores the non-linear hidden impacts of income inequality on health outcomes in emerging economies, though past studies have to ignore the non-linear relationships. This study will bring new health insights for authorities and policymakers in an era of a pandemic. The study will offer appropriate policy suggestions for emerging and developing economies. To that end, we have applied advanced panel data techniques such as panel ARDL-PMG, DOLS, and FMOLS. The organization of the study is as follows. The model and methods are discussed in section Model, methods, and data, and the results in section Results and discussion. Finally, we provide a conclusion in section Conclusion and implications.

## Model, Methods, and Data

Our main motive is to capture the impact of income inequality on health. To achieve that goal, we have borrowed a model from Bakkeli (14).


(1) $$\begin{array}{l} \begin{array}{cc} \text{Healt}{\text{h}}_{\text{it}} & =\;{\text{ω}}_{0}+\;{\text{φ}}_{1}\text{Gin}{\text{i}}_{\text{it}}+{\text{φ}}_{2}\text{GD}{\text{P}}_{\text{it}}+{\text{φ}}_{3}\text{Unemploymen}{\text{t}}_{\text{it}} \end{array}\\ \;\begin{array}{c} +\;{\text{φ}}_{4}\text{Educatio}{\text{n}}_{\text{it}}+{\text{ε}}_{\text{it}} \end{array} \end{array}$$

Specification (1) is the health function that relies on income inequality (Gini), gross domestic product (GDP), unemployment, and education. We used life expectancy as a proxy of health. To convert this equation into panel ARDL-PMG, we need to, respectively. equation (1) into error correction format as described below:


(2) $$\begin{array}{l} \Delta \text{Healt}{\text{h}}_{\text{it}}=\\ \;\;{\text{ω}}_{0}+\;{\sum^{\text{​}}}_{\text{k}=1}^{\text{n}}\beta_{1\text{k}}\Delta \text{Healt}{\text{h}}_{\;\text{it}-\text{k}}+\overset{\text{k}=0}{\underset{\text{n}}{\sum^{\text{​}}}}\beta_{2\text{k}}\Delta \text{Gin}{\text{i}}_{\text{it}-\text{k}}\\ \;+\;{\sum^{\text{​}}}_{\text{k}=1}^{\text{n}}\beta_{3\text{k}}\Delta \text{GD}{\text{P}}_{\text{it}-\text{k}}+\overset{\text{k}=0}{\underset{\text{n}}{\sum^{\text{​}}}}\beta_{4\text{k}}\Delta \text{Unemploymen}{\text{t}}_{\text{it}-\text{k}}\\ \;+{\sum^{\text{​}}}_{\text{k}=0}^{\text{n}}\beta_{5\text{k}}\Delta \text{Educatio}{\text{n}}_{\text{it}-\text{k}}+{\text{ω}}_{1}\text{Healt}{\text{h}}_{\text{it}-1}\\ \;+\;{\text{ω}}_{2}\text{Gin}{\text{i}}_{\text{it}-1}+{\text{ω}}_{3}\text{GD}{\text{P}}_{\text{it}-1}+{\text{ω}}_{4}\text{Unemploymen}{\text{t}}_{\text{it}-1}\\ \;+\;{\text{ω}}_{5}\text{Educatio}{\text{n}}_{\text{it}-1}+{\text{ε}}_{\text{it}} \end{array}$$

Arrangement (2) has now become panel ARDL-PMG of Pesaran et al. (22) and Pesaran et al. (23). In equation, subscript i = 1,2,.,5 denotes the cross-sectional dimension; t is the time dimension, and k is the corresponding lag order. The method is superior to most techniques because it provides both the short and long-run estimates by analyzing a single equation. Moreover, we can add a mixture of I(0) and I(1) variables into our panel ARDL-PMG model. Furthermore, it is an efficient technique even if the sample size is small. The asymmetric model is performing better than the symmetric model. The NARDL approach is more flexible to the cointegration dynamics between concern variables. Such types of approaches are also explored non-linear hidden impacts of income inequality and health outcomes. However, in this study, we have also applied the non-linear panel ARDL-PMG model, and for that purpose, we have decomposed the variables of Gini into its positive and negative components by using the partial sum procedures as shown below:


$$\begin{array}{cc} \text{Gin}{\text{i}}^{+}{}_{\text{it}} & =\overset{\text{n}=1}{\underset{\text{t}}{{\sum^{\text{​}}}^{\text{​}}}}\text{Gin}{\text{i}}^{+}{}_{\text{it}}=\overset{\text{n}=1}{\underset{\text{t}}{{\sum^{\text{​}}}^{\text{​}}}}\text{max }(\text{Gin}{\text{i}}^{+}{}_{\text{it}},\;0) \end{array}\;(3\text{a})$$

$$\begin{array}{rc} {\text{Gin}{\text{i}}^{-}}_{\text{it}} & =\overset{\text{t}}{\underset{\text{n}=1}{\sum }}{{\text{Gini}}^{-}}_{\text{it}}=\overset{\text{t}}{\underset{\text{n}=1}{\sum }}\mathrm{mtextn}\;\left({{\text{Gini}}^{\;-}}_{\text{it}},\;0\right)\;\left(3\text{b}\right) \end{array}$$

The positive shocks in the series are represented by Gini^+^, whereas Gini represents the negative shocks in the series^−^. Next, we replace these partial sum variables in the place of original variables in equation (2), and the outcome of this action is shown below:


(4) $$\begin{array}{l} \Delta \text{Healt}{\text{h}}_{\text{it}}\;=\;\;\omega_{0}+\;\overset{\text{n}}{\underset{\text{k}=1}{\sum^{\text{​}}}}{\text{β}}_{1\text{k}}\text{ΔHealt}{\text{h}}_{\text{it}-\text{k}}+\;\overset{\text{n}}{\underset{\text{k}=0}{\sum^{\text{​}}}}{\text{β}}_{2\text{k}}\text{Gin}{\text{i}}^{+}{}_{\text{it}-\text{k}}\\ \;+\;\overset{\text{n}}{\underset{\text{k}=0}{\sum^{\text{​}}}}{\text{δ}}_{3\text{k}}\text{Gin}{\text{i}}^{-}{}_{\text{it}-\text{k}}+\;\overset{\text{n}}{\underset{\text{k}=0}{\sum^{\text{​}}}}{\text{β}}_{6\text{k}}\text{GD}{\text{P}}_{\text{it}-\text{k}}+\\ \;\overset{\text{n}}{\underset{\text{k}=0}{\sum^{\text{​}}}}{\text{β}}_{7\text{k}}\text{Unemploymen}{\text{t}}_{\text{it}-\text{k}}+\overset{\text{n}}{\underset{\text{k}=0}{\sum^{\text{​}}}}{\text{β}}_{7\text{k}}\text{Educatio}{\text{n}}_{\text{it}-\text{k}}\\ \;+\;\omega_{1}\text{Healt}{\text{h}}_{\text{it}-1}+\;\omega_{2}\text{Gin}{\text{i}}^{+}{}_{\text{it}-1}+\;\omega_{3}\text{Gin}{\text{i}}^{-}{}_{\text{it}-1}\\ \;+\;\omega_{6}\text{GD}{\text{P}}_{\text{it}-1}+\omega_{7}\text{Unemploymen}{\text{t}}_{\text{it}-1}\\ \;+\;\omega_{7}\text{Educatio}{\text{n}}_{\text{it}-1}+\;{\text{ε}}_{\text{it}} \end{array}$$

Equation (4) is known as the panel NARDL-PMG model proposed by Shin and Greenwood (24), and this is an advanced form of the linear ARDL-PMG. Therefore, non-linear panel PMG can be dealt with the estimation procedure and diagnostic test of the panel ARDL-PMG. Moreover, the cointegration test and critical values are also the same for both models.

For robust analysis, this study is used pooled-OLS (P-OLS), dynamic ordinary least squares (DOLS), and fully modified ordinary least squares (FMOLS) estimators in analysis. The DOLS and FMOLS are highly efficient in handling the issue of serial correlations in the error terms and endogeneity among regressors. The FMOLS is considered one of the non-parametric approaches that control autocorrelation and endogeneity problems Pedroni (25), whereas the DOLS approach eliminates the by adding leads and lags of the explanatory variables (26). At the same time, DOLS is one of the parametric approaches and gives better results in small samples (27). Particularly, the DOLS method can handle cross-sectional dependence (CD) based on the gaining of country-specific coefficients and produce unbiased, efficient, and consistent estimates. Pedroni (25) authors noted that the panel DOLS is less biased than the POLS and FMOLS estimators in small samples. At the same time, the DOLS estimator has better sample properties than the POLS and FMOLS estimators. The Dumitrescu and Hurlin (DH) causality test considers heterogeneity and cross dependence, while it produces a robust estimate for small data.

To do empirical analysis, we collect cross-sectional data for five Asian emerging countries, namely China, India, Japan, Indonesia, and Turkey. The data set is missing of our focused variables which were observed from 1991 to 2019. The missing data is completed through the extrapolation method. To measure health outcomes, life expectancy at birth, total (years) variables are employed. The elements used in our analysis are the Gini index which measures income inequality, GDP per capita (current US$), unemployment, total (% of the total labor force), the average year of schooling (education). The data set was constructed from the (28, 29).

## Results and Discussion

First of all, we apply three different panel unit root tests to confirm whether our variables are stationary at level or first difference because the application of NARDL requires that none of the variables in the model should be I (2). For that purpose, we have applied three-panel unit root tests Levin, Lin, and Chin (LLC), Pesaran and Shin (30), and ADF-Fisher. These tests are reported in Table 1, which states that most of the variables are stationary at a level with all three tests except GDP, unemployment, and education. After confirming that our variables are either I(0) or I(1), we can now apply NARDL, and a maximum of two lags are imposed as our data is annual. For selecting an appropriate number of lags, we have applied Akaike Information Criterion (AIC).

**Table 1:** Panel unit root testing.

	**LLC**			**IPS**		
	**I(0)**	**I(1)**	**Decision**	**I(0)**	**I(1)**	**Decision**
Health	−6.420***		I(0)	−2.094**		I(0)
Gini	−2.184*		I(0)	−0.148	−4.456***	I(1)
GDP	−0.839	−3.981***	I(1)	−0.374	−4.628***	I(1)
Unemployment	−1.182	−4.112***	I(1)	−1.236	−2.965***	I(1)
Education	−0.839	−3.170***	I(1)	−0.548	−5.596***	I(1)

*** *p <0.01*;

** *p <0.05; and*

* *p <0.1*.

To investigate the relationship between income inequality and health outcomes, we have decided to apply the linear and non-linear panel ARDL-PMG as our baseline models, reported in Table 2. Then, to check the robustness of our results, we have applied linear and non-linear POLS, FMOLS, DOLS reported in Table 3. Lastly, we have also performed a panel causality test, and its results are presented in Table 4. Table 2 provide the short-run and long-run estimates of our baseline models. The results of cointegration tests, i.e., ECM(−1) and Kao-cointegration, are also reported in Table 2. First, we see whether the cointegration exists between our long-run variables or not. Relying on the significant values of Kao and Chiang (26) (−1), we can confirm that the long-run relationship between health outcome, income inequality, GDP, unemployment, and education is valid in both the models, i.e., ARDL and NARDL.

**Table 2:** ARDL-PMG and NARDL-PMG estimates of life expectancy.

	**ARDL-PMG**				**NARDL-PMG**			
**Variable**	**Coefficient**	**S.E**	***t*-Stat**	Prob.*	**Coefficient**	**S.E**	***t*-Stat**	Prob.*
**Long-Run**
GINI	−0.151*	0.030	4.949	0.000				
GINI_POS					−0.168***	0.030	5.585	0.000
GINI_NEG					−0.197***	0.053	3.721	0.000
GDP	0.007	0.004	1.585	0.117	0.101	0.175	0.578	0.565
UNEMPLOYMENT	−0.026***	0.003	7.516	0.000	0.111	0.159	0.700	0.486
EDUCATION	0.050***	0.004	13.32	0.000	0.534	0.709	0.753	0.453
**Short-Run**
COINTEQ01	−0.320**	0.145	2.133	0.045	−0.270**	0.035	7.721	0.000
D(GINI)	0.080	0.112	0.715	0.477				
D[GINI(−1)]	0.039	0.062	0.626	0.533				
D(GINI_POS)					−0.031	0.037	0.834	0.406
D(GINI_NEG)					0.053	0.048	1.100	0.274
D(GDP)	0.003	0.003	0.903	0.369	0.004**	0.002	2.000	0.040
D[GDP(−1)]	0.003***	0.001	2.439	0.017				
D(UNEMPLOYMENT)	0.000	0.001	0.195	0.846	0.001	0.001	0.967	0.336
D[UNEMPLOYMENT(−1)]	0.001*	0.001	1.785	0.079				
D(EDUCATION)	0.001	0.001	0.661	0.511				
D[EDUCATION(−1)]	−0.001	0.002	0.487	0.628	0.000	0.001	0.176	0.861
C	0.002	0.090	0.020	0.984	0.001	0.002	0.615	0.540
**Diagnostics**
Log-Likelihood	836.5				824.1			
Kao-Cointergation	2.878***				2.012***			
Wald-LR					4.456**			
Wald-SR					1.325			

*** *p <0.01*;

** *p <0.05; and*

* *p <0.1*.

**Table 3:** Robustness check.

	**P-OLS**	**FM-OLS**	**D-OLS**	**P-OLS**	**FM-OLS**	**D-OLS**
Gini	−0.175***	−0.090***	−0.050***			
	(6.690)	(6.320)	(8.402)			
Gini_pos				−0.110***	−0.040***	−0.050***
				(5.780)	(14.94)	(7.330)
Gini_neg				−0.282***	−0.320***	−0.080***
				(6.400)	(8.980)	(10.23)
GDP	0.019***	0.010***	0.030***	0.027***	0.010***	0.020***
	(5.110)	(13.32)	(8.703)	(7.580)	(7.690)	(3.410)
Unemployment	−0.002*	−0.010***	−0.010***	−0.004***	−0.0001***	−0.0001***
	(1.810)	(5.300)	(4.010)	(2.920)	(7.700)	(5.480)
Education	0.023***	0.020***	0.020***	0.026***	0.020***	0.020***
	(10.15)	(45.30)	(6.320)	(9.640)	(9.540)	(8.640)

*** *p <0.01*;

**p <0.05; and

* *p <0.1*.

**Table 4:** Panel symmetric and asymmetric causality results.

**Null hypothesis**	**W-Stat**.	**Zbar-Stat**.	**Prob**.	**Null hypothesis**	**W-Stat**.	**Zbar-Stat**.	**Prob**.
GINI → LE	27.52	23.28	0.000	GINI_POS → LE	14.69	18.382	0.000
LE → GINI	4.432	2.052	0.040	LE → GINI_POS	3.794	3.653	0.000
GDP → LE	44.60	38.986	0.000	GINI_NEG → LE	2.14	1.418	0.156
LE → GDP	5.683	3.202	0.001	LE → GINI_NEG	1.715	−0.446	0.657
UNEMPLOYMENT → LE	4.095	1.742	0.082	GDP → LE	2.519	1.948	0.051
LE → UNEMPLOYMENT	7.562	4.929	0.000	LE → GDP	2.064	1.33	0.184
EDUCATION → LE	16.25	12.917	0.000	UNEMPLOYMENT → LE	5.487	5.988	0.000
LE → EDUCATION	5.729	3.244	0.001	LE → UNEMPLOYMENT	4.945	5.25	0.000
GDP → GINI	4.047	1.698	0.090	EDUCATION → LE	28.22	36.925	0.000
GINI → GDP	4.160	1.802	0.072	LE → EDUCATION	2.318	1.675	0.094
UNEMPLOYMENT → GINI	1.713	−0.448	0.654	GINI_NEG → GINI_POS	9.285	8.652	0.000
GINI → UNEMPLOYMENT	4.446	2.065	0.039	GINI_POS → GINI_NEG	1.713	−0.448	0.654
EDUCATION → GINI	1.579	−0.57	0.568	GDP → GINI_POS	6.524	7.342	0.000
GINI → EDUCATION	7.785	5.135	0.000	GINI_POS → GDP	2.422	1.799	0.072
UNEMPLOYMENT → GDP	2.233	0.03	0.976	UNEMPLOYMENT → GINI_POS	2.848	2.375	0.018
GDP → UNEMPLOYMENT	5.446	2.984	0.003	GINI_POS → UNEMPLOYMENT	6.997	7.982	0.000
EDUCATION → GDP	2.285	0.078	0.937	EDUCATION → GINI_POS	4.581	4.717	0.000
GDP → EDUCATION	3.159	0.882	0.378	GINI_POS → EDUCATION	0.511	−0.784	0.433
EDUCATION → UNEMPLOYMENT	5.577	3.105	0.002	GDP → GINI_NEG	1.579	−0.57	0.568
				GINI_NEG → GDP	2.348	1.702	0.089
UNEMPLOYMENT → EDUCATION	1.458	−0.682	0.495	GINI_NEG → UNEMPLOYMENT	4.257	4.279	0.000
				EDUCATION → GINI_NEG	3.100	0.870	0.360
				GINI_NEG → EDUCATION	6.215	6.926	0.000
				UNEMPLOYMENT → GDP	2.083	1.355	0.176
				GDP → UNEMPLOYMENT	3.726	3.591	0.000
				EDUCATION → GDP	0.800	−0.39	0.696
				GDP → EDUCATION	2.239	1.568	0.117
				EDUCATION → UNEMPLOYMENT	4.934	5.234	0.000
				UNEMPLOYMENT → EDUCATION	0.386	−0.954	0.340

***p <0.01; **p <0.05; and *p <0.1.

From Table 2, we see that the coefficient estimate of GINI is negatively significant, which suggests that a 1% rise in income inequality in Asian economies reduces life expectancy by 0.151%. In the non-linear model, the positive change in income inequality (GINI_POS) is negative, and the negative change in the income inequality (GINI_NEG) is also negative. From these findings, we confirm that a 1% rise in income inequality causes the life expectancy to fall by 0.168%, and a 1% reduction in income inequality causes the life expectancy to rise by 0.197%. The non-linear results also confirm the asymmetric impact of income inequality on health outcomes in emerging Asian economies. The significant estimate of WALD-LR also confirms the long-run asymmetric effects between GINI_POS and GINI_NEG reported in Table 2. Both linear and non-linear estimates complement each other, and the results are as per expectations. Now, if we turn our attention to the robust models, the estimates of GINI appeared to be negatively significant irrespective of the estimation technique. Similarly, in the robust asymmetric models, the sign of the estimates attached to GINI_POS and GINI_NEG are the same, just like our asymmetric baseline model. Hence, we can say that baseline results are robust.

This finding is consistent with Qasim et al. (16), who infers that income inequality adversely affects human development in Pakistan. This outcome is not surprising for emerging Asian economies because Tibber et al. (15) found a similar conclusion in a systematic review. In general, we can say that income inequality is not good for the overall health status of nations. Health is the most primary concern not only for individuals but for governments and policymakers. A healthy mind and body can play a positive role in achieving long-term economic goals and becoming an asset for society; however, a sick mind and body can become a liability. Health is not just the absence of physical illness, but a person is considered healthy if he is mentally fit and actively contributing to the well-being of society (1). According to Black (31) the health status of people, who belongs to different socioeconomic status, varies drastically. The health status of the poor and deprived people is below average, whereas the affluent socioeconomic class of the society enjoys much better health facilities and status, particularly in the developing and emerging economies.

After the financial crisis of the year 2008–2009, people started to raise their voices against income inequality and its related problems because during the period 2009–2012, the income of the top 1% richest people in the USA increased drastically while the income of other 99% increased marginally. Such a drastic difference in the income of the two factions of the society raised eyebrows of many. On one side, economic inequality will negatively affect the mental health of the lower-income class due to a sense of deprivation. On the other side, the number of social capital facilities for this class will also be reduced. They also do not have access to better education facilities; hence, the level of awareness among them vis-à-vis their health status is very low. Furthermore, as the economic disparity between the rich and poor class increases, it also increases the difference between their socioeconomic status and related indicators such as education, living standards, health, and the problem is more severe in the emerging economies (2, 32). Our findings also supported the arguments given by the previous researchers and confirmed that economic disparity negatively impacts the health status of the people.

Among the control variables, the estimates of GDP are insignificant in the baseline linear and non-linear models, whereas significant and positive in all the robust models, implying that as the level of affluence in the economy goes up, life expectancy also increases. The estimated coefficient of UNEMPLOYMENT is negatively significant in the linear baseline model, whereas positive but insignificant in the non-linear baseline model. Similarly, UNEMPLOYMENT estimates are negatively significant in the linear and non-linear models with all three estimation techniques in the robust models. These findings suggest that a decline in the level of unemployment will have a positive impact on health outcomes. However, the size of the estimates is too small in the baseline, and the robust models suggest that though the decline in the unemployment rate improves the health status of the people in emerging Asian economies, the magnitude of these effects is not that large. Finally, the EDUCATION variable appeared to be significant and positive in the linear ARDL-PMG model and insignificant in the NARDL-PMG model. On the other hand, in the linear and non-linear robust model, the EDUCATION estimates were positively significant with any estimation techniques. We can deduce that education can make people more cautious about their health status, and consequently the life expectancy increases.

Table 2 also provides the short-run result, and from seeing them, we can say that the short-run estimates of most of the variables provided are mi**xe**d, to say the least. The detailed results of the causal analysis are provided in Table 4. However, for our readers' interest, we discuss some important results, i.e., we observe the bi-directional causality between GINI↔LE, GDP↔LE, EDUCATION↔LE, UNEMPLOYMENT↔LE, and GINI_POS↔LE.

## Conclusion and Implications

The health of the population is a significant economic concern for the world. It plays a central role in the development process. Therefore, this study empirically examined the asymmetric impact of income inequality on health status in Asian emerging economies. We found that income inequality has a statistically negative significant influence on health in the symmetric model in the long run. Similarly, asymmetric findings of income inequity have deviated from the symmetric model in the long run. A positive change in income inequity negatively impacts health, while a negative change in income inequity positively impacts health in the long run in emerging Asian economies. Findings show that income inequity has no impact on health in symmetric and asymmetric models in the short run. The robust regression models of study have obtained similar findings of long-run in FMOLS and DOLS. Regarding control variables, unemployment is negative, and education positively impacts health in the long run. Unemployment has an unfavorable impact on health in the long run. Education is one key determinant of health outcomes, as education has favorable impacts on health in the long run.

### Policy Implications

Regarding implications, the authorities can improve the health of the population via the redistribution of income. Policymakers should also reduce urban-rural inequality for better population health. Government should provide better health facilities to poor people. Asian policymakers should focus on strengthening the basic health care systems in their countries to reduce the adverse effect of income inequality on health. Asian governments give special attention to reducing health inequality. The governments and the international community should pay more attention to the consequences of their economic policies on income and health inequality to improve population health. A well-designed “National Health Insurance Scheme” in emerging Asian economies can significantly alleviate inequalities in health.

### Limitations and Future Directions

This research could not found a relationship between income inequality, mental and physical health outcomes. The study could not incorporate the COVID-pandemic in empirical analysis. There is a need for further work to scrutinize the impact of income inequality on mental and physical health at micro-level data. This kind of work will help explain how income inequality impacts health outcomes for different population sectors. Such studies will further improve our implications on health. Finally, future studies on the link between income inequality and mental and physical health might use other measures of income inequality such as Gini coefficient, Palma ratio, Decile ratio, Theil Index, Lorenz curve, and Log Mean Deviation. The upcoming studies assessing the key determinants of income-related health inequalities during the COVID-19 pandemic are significant for redesigning appropriate policies.

## Data Availability Statement

The original contributions presented in the study are included in the article/supplementary material, further inquiries can be directed to the corresponding author/s.

## Author Contributions

SC: conceptualization, software, data curation, and writing—original draft preparation. BG: methodology, visualization, and investigation. Both authors contributed to the article and approved the submitted version.

## Funding

The paper was supported by the Project of Humanities and Social Sciences of Cultivation Plan for 1,0000 Middleaged and Young Backbone Teachers in Higher Education Institutions in Guangxi (2020QGRW016), the Natural Science Foundation of Guangxi Province of China (AD20159052).

## Conflict of Interest

The authors declare that the research was conducted in the absence of any commercial or financial relationships that could be construed as a potential conflict of interest.

## Publisher's Note

All claims expressed in this article are solely those of the authors and do not necessarily represent those of their affiliated organizations, or those of the publisher, the editors and the reviewers. Any product that may be evaluated in this article, or claim that may be made by its manufacturer, is not guaranteed or endorsed by the publisher.
